# Management of a patient with common variable immunodeficiency and hepatopathy

**DOI:** 10.1186/s13223-023-00799-y

**Published:** 2023-06-05

**Authors:** Lea Grümme, Hendrik Schulze-Koops

**Affiliations:** Division of Rheumatology and Clinical Immunology, Department of Medicine IV, LMU Clinic Munich, Munich, Germany

**Keywords:** CVID, Common variable immunodeficiency, Hepatitis, Hepatopathy

## Abstract

**Background:**

Common variable Immunodeficiency (CVID) is a primary immunodeficiency disorder and the most common form of severe antibody deficiency. Both children and adults are affected and clinical manifestations vary widely. Often, CVID manifests with infections, autoimmune phenomena or chronic lung disease, but it also frequently affects the liver. The differential diagnoses of hepatopathies in CVID patients are diverse and the characteristics of CVID patients often make it difficult to determine the correct diagnosis.

**Case presentation:**

We present the case of a 39-year-old patient with CVID and elevated liver enzymes, nausea and unintended weight loss, who was referred to our clinic with the suspected diagnose of autoimmune hepatitis or immunoglobulin-induced hepatopathy. Prior, the patient had undergone an extensive diagnostic work-up including liver biopsy but viral hepatitides had only been investigated serologically – with negative antibody results. We searched for viral nucleic acid by polymerase chain reaction and detected hepatitis E virus-RNA. Antiviral therapy was started and the patient recovered quickly.

**Conclusion:**

Hepatopathies in CVID patients are common with a broad spectrum of possible causes. While treating CVID patients, the distinct diagnostic and therapeutic requirements of the CVID patients should be closely considered and diagnosed by the appropriate measures.

## Background

Common variable Immunodeficiency (CVID) is a primary immunodeficiency disorder characterized by impaired B-cell differentiation and defective immunoglobulin (Ig) synthesis. This results in a decreased serum concentration of immunoglobulins, usually IgG, often IgA and sometimes IgM. Both children and adults are affected, with most patients being diagnosed between the age of 20 and 40 due to frequently occurring delayed diagnosis [1, 2]. CVID is the most common form of severe antibody deficiency [1]. The manifestations of CVID vary widely, hence the term “variable” in the name of the disease. Patients have a significantly increased risk of developing non-Hodgkin’s lymphoma and the most common clinical manifestations of CVID are infections, autoimmune phenomena, and chronic lung disease. However, liver disease is also present in approximately 10% of CVID patients [3–5]. These patients often show elevated alkaline phosphatase and liver dysfunction [4]. Often, anicteric cholestasis and/or portal hypertension are also present [6]. Patients may otherwise be asymptomatic or have complaints of fatigue, jaundice, pruritus, ascites, edema, nausea, vomiting, esophageal varices, and hepato- or splenomegaly [7]. Possible differential diagnoses of liver disease in patients with CVID include infections, autoimmune reactions, lymphoproliferation, malignancies, granulomas, infiltration of inflammatory cells, and intrahepatic biliary obstruction [8]. For further diagnostic workup and initiation of adequate therapy of the liver involvement, the special requirements of CVID patients must be taken into account. This leads to specific diagnostic and therapeutic consequences in the treatment of hepatopathies in CVID patients.

## Case presentation

We present the case of a 39-year-old patient with CVID and elevated liver enzymes, nausea and unintended weight loss.

The patient had been diagnosed with CVID in 03/2006 due to recurrent respiratory infections, splenomegaly, tricytopenia including mild anemia, thrombocytopenia, and lymphocytopenia (moderate CD3 T, severe CD19 B and CD56 NK cell deficiency) and decreased immunoglobulins (IgG 1.5 g/l (reference: 7–16 g/l), IgA: <0.01g/l (reference: 0.7-4 g/l), IgM: <0.01 g/l (reference: 0.4–2.3 g/l). In 2013, he developed a predominant lymphocyte-rich Hodgkin’s lymphoma (stage IA, remission after therapy with rituximab 8 years ago) and a chronic, polypous rhinosinusitis, which was treated with dupilumab 300 mg subcutaneously every 2 weeks since 05/2020. He did not smoke or drink alcohol. Since the CVID diagnosis, he had been treated with immunoglobulins. For the first 6 years after diagnosis, he received the immunoglobulins intravenously and since 2012 subcutaneously. He had always tolerated the immunoglobulin substitution well.

One year prior to admission to our clinic, he presented with elevated liver enzymes in a routine check-up at his general physician. He was transferred to a gastroenterologist and the diagnostic procedures included multiple hepatitis serologies, which did not reveal any abnormal findings. A therapy with a locally acting cortisone was initiated (budenoside 6 mg/day; a dosage of 9 mg/day had not been well tolerated) but the liver enzymes remained elevated. After six months, the patient developed nausea and unintended weight loss. A liver biopsy was performed. The biopsy revealed liver tissue with inflammation intraacinar and portal, a slight cholestasis and a moderate fibrosis. Infiltrates of Hodgkin’s lymphoma were not evident. A suspected diagnosis of “drug-toxic liver damage of the mixed hepatic-cholestatic subtype in the sense of autoimmune liver disease” was stated. It was suspected that the hepatopathy could have been induced by the administered immunoglobulins. Therefore, immunoglobulin substitution was stopped. Hence, the patient was transferred to our outpatient clinic in February 2021 with the differential diagnoses “autoimmune hepatitis or immunoglobulin-induced hepatopathy”.

Upon his first visit, he presented in a reduced general condition and a slim nutritional status. His treatment consisted of budenoside 6 mg/day and duplimumab 300 mg subcutaneously every two weeks. He reported that he had unintentionally lost 20 kg of weight in the last 6 months and felt significantly weakened. He experienced frequent nausea and vomiting, especially in the morning. He had no other gastrointestinal symptoms. He had splenomegaly, while there was no evidence of hepatomegaly. The patient did not show any liver-specific symptoms such as jaundice or other pathognomonic skin signs.

**Fig. 1:**
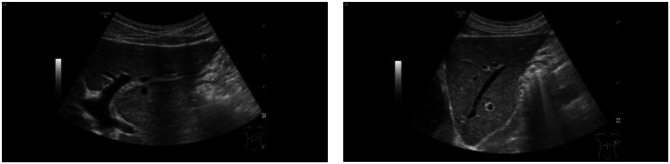
Abdominal ultrasound showed no signs of fibrosis or cirrhosis of the liver with smooth organ contour, acute-angled lower rim, homogeneous echonormal internal reflex pattern, inconspicuous vascular architecture and no focal lesions

In the initial laboratory examination on 02/03/2021, liver enzymes were elevated (aspartate aminotransferase (GOT/AST): 341 U/l (reference value: ≤ 49 U/l), alanine aminotransferase (GPT/ALT): 895 U/l (reference value: ≤ 49 U/l), gamma-glutamyltransferase (gamma-GT): 665 U/l (reference value: ≤ 59 U/l), alkaline phosphatase: 408 U/l (reference value: 40–130 U/l), total bilirubin: 2.0 mg/dl (reference value: ≤ 1.2 mg/dl) (Table 1). Consistent with a currently untreated CVID, immunoglobulins were decreased (IgG 2.21 g/l (reference value: 7–16 g/l), IgA: <0.05 g/l (reference value: 0.7-4 g/l), IgM: <0.05 g/l (reference value: 0.4–2.3 g/l)) (Table 1). Albumin levels were normal at the time of low IgG (Table 1). Abdominal ultrasound showed no signs of fibrosis or cirrhosis of the liver (Fig. 1).

**Table 1:** Extract from the patient’s laboratory results between February 2021 and June 2021

	unit	material	reference	results					
			value						
date				02/03/2021	02/11/2021	02/24/2021	03/11/2021	04/15/2021	06/04/2021
total bilirubin	mg/dl	serum	≤ 1,2	2		2,3		0,5	
aspartate aminotransferase (GOT/AST)	U/l	serum	≤ 49	341	266	242	209	62	84
alanine aminotransferase (GPT/ALT)	U/l	serum	≤ 49	895	810	528	406	84	142
gamma-glutamyltransferase (gamma − GT)	U/l	serum	≤ 59	665	648	593	480	145	97
alkaline phosphatase	U/l	serum	40–130	408	392			207	250
immunoglobulin G (IgG)	g/l	serum	7,00–16,00	2,21	1,93	4,28	5,29	9,5	8,8
immunoglobulin A (IgA)	g/l	serum	0,70–4,00	< 0,05	< 0,10		< 0,05	< 0,05	< 0,05
immunoglobulin M (IgM)	g/l	serum	0,40–2,30	< 0,05	< 0,10		< 0,05	< 0,05	< 0,05
albumin	g/dl	serum	3,5–5,2	4,1	4				

Considering the severe gastrointestinal symptoms with almost daily morning sickness, significant weight loss and elevated transaminases, we tested for viral hepatitides. In addition to serological testing, we also performed a testing via polymerase chain reaction (PCR), since serological testing is not sufficiently reliable in CVID patients [1]. In the PCR, the HCV-RNA, HAV-RNA and HBV-DNA were negative, while the HEV-RNA turned out positive. Except the CVID, the patient had no other risk factor for HEV. The markers of autoimmune hepatitis were negative, however, the limited value in CVID patients must be pointed out [9] (negative: ANA (antinuclear antibody), AMA (anti-mitochondrial antibody), AMA-M2-Ab (anti-mitochondrial antibody, branched-chain alpha-keto acid dehydrogenase complex), SMA (smooth muscle antibody), LKM-Ab (anti-liver-kidney microsome antibody), LKM-1-Ab (CCp450) (anti-liver-kidney microsome antibody or CYP2D6 antibody), and SLA-Ab (anti-soluble liver antigen antibody)).

**Fig. 2:**
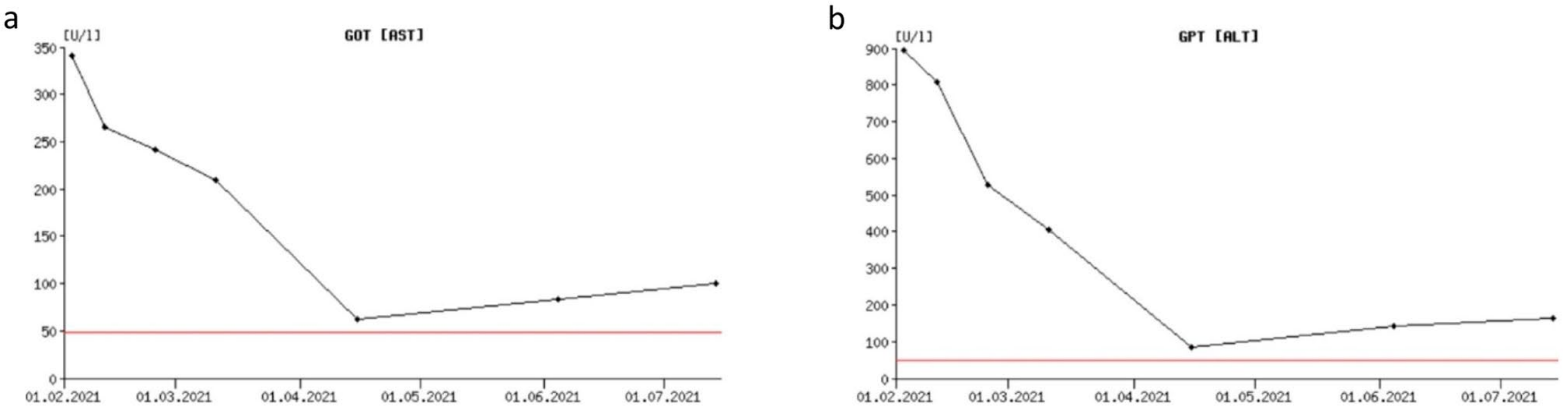
a: Time course of aspartate aminotransferase (GOT/AST) levels. Start of anti-viral therapy (Ribavirin) with 200 mg 1-0-1 on 02/09/2021, increase of dosage to 400 mg 1-0-1 on 03/11/2021. Negative PCR detection for HEV-RNA on 06/07/2021. b: Time course of alanine aminotransferase (GPT/ALT) levels. Start of anti-viral therapy (Ribavirin) with 200 mg 1-0-1 on 02/09/2021, increase of dosage to 400 mg 1-0-1 on 03/11/2021. Negative PCR detection for HEV-RNA on 06/07/2021

We concluded that the patient’s hepatopathy was more likely due to hepatitis E infection than to immunoglobulin-induced origin, as it had been previously suspected. We started a therapy with ribavirin (initial dose: 200-0-200 mg, then 400-0-400 mg because the drug was well tolerated). After 12 weeks the liver enzymes had decreased significantly (GOT/AST: 84 U/l (reference value: ≤ 49 U/l), GPT/ALT: 142 U/l (reference value: ≤ 49 U/l), gamma-GT: 97 U/l (reference value: ≤ 59 U/l)) (Table 1; Fig. 2). Clinical symptoms had also improved: The patient described that the frequency of the morning sickness had significantly decreased and that he had already regained weight. About 6 months after starting the anti-viral medication, the HEV-RNA was no longer detectable by PCR, while the transaminases showed a slight increase (Table 1; Fig. 2). Despite the slight increase of the transaminases, we then terminated the anti-viral therapy due to no longer detectable HEV-RNA and clinical improvement of the patient.

## Discussion

In the treatment of CVID patients, it is important to keep the special requirements and circumstances of the patients in mind.

At first presentation, the patient described that he had been suffering from severe gastrointestinal symptoms for more than 6 months including nausea and weight loss, and has also had significantly elevated transaminases for about a year. This is common in CVID patients, as approximately 10% of all CVID patients develop liver involvement [3–5]. The differential diagnoses of hepatopathies in CVID patients are diverse. In our patient, the suspected diagnoses at the time of referral to our clinic were autoimmune hepatitis or immunoglobulin-induced hepatopathy.

In the literature, elevated transaminases have been described after administration of intravenous immunoglobulins (IvIg) [10]. However, they were rather mildly elevated and the increase was self-limiting after a few days [10]. In addition, hepatitis itself, as well as symptoms such as nausea or vomiting, are described as possible side effects of IvIg [11]. To the best of our knowledge, such a severe and prolonged toxic drug reaction to immunoglobulin replacement therapy (as it would have been the case in our patient) has not been described so far.

In rare cases, the liver involvement presents as a disease resembling autoimmune hepatitis (AIH) [9, 12]. AIH is an inflammatory liver disease characterized by elevated autoantibodies, hypergammaglobulinemia, and hepatocellular damage with lymphoplasmacytic infiltrate [9]. However, the diagnosis of AIH in CVID patients is difficult. The fulfillment of the diagnostic criteria (antibodies, elevated serum IgG level, absence of alternative diagnoses and compatible liver biopsy) is considerably more difficult due to the decreased serum IgG level and unreliable serology. In addition, the liver biopsy is not always conclusive in AIH, as it may overlap with other entities [9, 13]. Therefore, indication for an autoimmune cause is mainly the response to immunosuppressive therapy [9, 14]. Autoimmune phenomena occur frequently in CVID patients, most likely due to impaired intrinsic immune regulation [15]. The most common clinical manifestations of autoimmunity in CVID patients are autoimmune hemolytic anemia (AIHA) and immune thrombocytopenia (ITP) [16].

Cases have also been described in which AIH was most likely triggered by a previous viral infection [14, 17]. In these cases, the autoimmune hepatitis persisted after successfully treating the viral hepatitis, suggesting that the immunopathogenic mechanisms of liver disease continued even without the ongoing presence of the virus [17].

Prior to his presentation to us, the patient had already undergone extensive diagnostics including a liver biopsy. In addition, several examinations for viral hepatitides had already been performed by numerous physicians - however, only by investigating the hepatitis serology. This is not sufficiently reliable in CVID patients due to the deficient antibody production [9]. We were the first to perform a PCR examination on the patient. Only through PCR testing, we were able to diagnose the hepatitis E infection and initiate anti-viral therapy. This led to a rapid improvement of the patient’s symptoms and laboratory results.

Transmission of HEV usually occurs through contaminated food, water, blood transfusions, or mother-to-child transmission. The diagnosis can be confirmed serologically by antibody determination or by HEV RNA assay from stool or serum. Treatment of HEV depends on the patient’s immune status and disease stage (e.g., acute versus chronic). For most immunocompetent patients, treatment of acute HEV infection is mostly supportive, as the disease is usually mild and self-limiting. In the treatment of chronic infection, any immunosuppressive therapy should first be reduced and/or antiviral therapy initiated. There are no randomized trials of the use of ribavirin for the treatment of chronic HEV, however, several studies have suggested benefit [18].

Of note, with the splenomegaly, severe B-cell deficiency, Hodgkin’s lymphoma, and onset of clinical symptoms as a young adult, the patient could be diagnosed with late-onset CVID. In contrast, the more mild T-cell lymphopenia might tend to argue against a diagnosis of this subset of CVID. In any case, treatment would not have been different, with the consequence that serum testing for infections would have been compromised.

Physicians treating patients with CVID should be aware of the special circumstances and requirements of their patients. Most importantly, testing for antibodies is not sufficiently reliable in CVID patients due to the deficient antibody production. Therefore, both markers for autoimmune hepatitis and serologic evidence of infection are not sufficiently informative in CVID patients. It is therefore important to aim for direct pathogen detection by PCR when an infectious cause of hepatopathy is suspected in CVID patients. It seems likely that if our patient had been tested for viral hepatitides by PCR earlier and not only by hepatitis serology, the patient could have been treated adequately by antiviral therapy more quickly and invasive diagnostics, such as liver biopsy, could have been avoided. This is particularly relevant with regard to the patient’s significantly reduced quality of life for many months as well as possible long-term consequences due to the prolonged hepatitis [19].

## Conclusion

CVID is an immunodeficiency with a broad range of clinical manifestations. Hepatopathies in CVID patients are common and the correct diagnostic procedures are crucial. Thus, the special requirements in CVID patients, which entail special diagnostic and therapeutic consequences, should be closely considered while treating CVID patients.

## Data Availability

Not applicable.

## List of Abbreviations

AIH: Autoimmune hepatitis
AIHA: Autoimmune hemolytic anemia
AMA: Anti-mitochondrial antibody
AMA-M2-Ab: Anti-mitochondrial antibody, branched-chain alpha-keto acid dehydrogenase complex
ANA: Antinuclear antibody
CVID: Common variable Immunodefiency
gamma-GT: Gamma-glutamyltransferase
GOT/AST: Aspartate aminotransferase
GPT/ALT: Alanine aminotransferase
Ig: Immunoglobulin
ITP: Immune thrombocytopenia
IvIg: Intravenous immunoglobulins
LKM-Ab: Anti-liver-kidney microsome antibody
LKM-1-Ab (CCp450): Anti-liver-kidney microsome antibody or CYP2D6 antibody
PCR: Polymerase chain reaction
SLA-Ab: Anti-soluble liver antigen antibody
SMA: Smooth muscle antibody
